# An Exploration of Contextual Aspects that Influence Cardiovascular Disease Risks Perceived by Workers in a Small–Medium-Sized Workplace

**DOI:** 10.3390/ijerph17145155

**Published:** 2020-07-17

**Authors:** Jin Ah Kim, Won Ju Hwang, Juhye Jin

**Affiliations:** 1 Department of Nursing, Gyeongju University, 188, Taejong-ro, Gyeongju-si, Gyeongsangbuk-do 38065, Korea; kimja@khu.ac.kr; 2 College of Nursing Science, East-West Nursing Institute, Kyung Hee University, Seoul 02447, Korea; 3 Department of Nursing, Korea National University of Transportation, Chungbuk 27909, Korea

**Keywords:** small–medium business, cardiovascular diseases, risk factors, occupational health, qualitative research

## Abstract

Contextual factors are associated with risks of cardiovascular disease (CVD) perceived by personnel employed in small–medium-sized workplaces. In an ecological model, data collection and analysis were undertaken, stratified by intrapersonal, interpersonal, and organizational contexts of blue-collar workers. Data were collected in face-to-face (*n* = 36) and focus group (*n* = 4) interviews and subjected to qualitative content analysis, to generate three main themes, 10 generic categories and 18 sub-categories. At the intrapersonal level, “physical burden”, “burn out due to overtime work”, “no time to take care of health because of family responsibility”, and “lack of recognition and knowledge of CVD risks” were derived from the individual interviews. At the interpersonal level, “stress of possible job losses”, “dislike of stigmatization of unhealthy persons”, “smoking and drinking to reduce relationship stress”, and “unhealthy work environment” differed by level of risk perception. “Preferred economic value” and “lack of understanding about importance of CVD management of an employer” emerged at the organizational level. Factors that influence CVD risks among workers in small–medium-sized business were present at the multiple levels. Therefore, healthcare providers in the field of occupational health should consider CVD risks in the context of blue-collar workers and organizational level for health-promotion programs.

## 1. Introduction

Small–medium-sized businesses with less than 300 employees [1] account for approximately 99.8% of all businesses in South Korea, and 85% of all employees work in small–medium-sized workplaces [2]. The 2017 Occupational Safety and Health Act does not impose any obligation on small–medium-sized businesses to appoint resident healthcare managers; even when healthcare managers are available, they can hold concurrent positions. Moreover, small businesses with fewer than 50 employees are not required to appoint healthcare managers [3]. Thus, the health of most workers in small–medium-sized companies are not systematically managed by dedicated healthcare management staff. Moreover, workers in small–medium-sized businesses who receive healthcare services have lesser opportunities for early disease screening and health-promotion education when compared to workers in larger businesses with more than 300 employees and resident healthcare managers. In this regard, the mortality rate from cardiovascular disease was higher in workers in small–medium-sized business than in larger businesses [4], which classifies workers in small–medium-sized businesses as a group vulnerable to healthcare management from a cardiovascular disease focus.

Previous studies conducted that—although factors affecting cardiovascular disease in small–medium-sized workplaces are influenced by multidimensional factors, ranging from individual lifestyles to organizational dimensions—interventional studies to prevent and manage this issue have been mainly conducted at the individual level [5,6]. However, according to McLeroy’s ecological model theory [7], factors affecting a person’s health can be influenced not only by the individual, but also by the environment surrounding the individual. Therefore, McLeroy emphasized, there must be an integrated consideration of personal factors and organizational factors. Moreover, recognizing disease risk factors can act as a major factor, which affects the health promotional behavior of individuals [8]. Therefore, it is important for healthcare staff who engage both workers and employers to identify the risk factors for cardiovascular disease in the practical context and apply them when planning health promotional programs to prevent and manage cardiovascular diseases in workers from small–medium-sized companies.

This study is theoretically based on the socioecological model, which takes a comprehensive approach to various levels of influencing factors to explore the contextual aspects of exposure risks to CVD perceived by workers in small–medium-sized workplaces. Besides the prevention of CVD in workers from small–medium-sized workplaces, the results of this study will contribute to establish evidence for effective disease management.

This study was conducted to explore the contextual aspects of CVD risk factors perceived by workers in a small–medium-sized workplace as: “How do members in a small–medium-sized workplace perceive the contextual aspects that influence cardiovascular disease risks?” Specific goals at the intrapersonal, interpersonal, and organizational levels based on socioecological models are as follows: First, to identify the contextual aspects of CVD risk factors perceived by individual members in a small–medium-sized workplace. Second, to investigate the contextual aspects of CVD risk factors among members that affect workers’ health in the small–medium-sized workplace. Third, to explore the contextual aspects of CVD risk factors in workers from the small–medium-sized workplace at the organizational level, as perceived by the employer and the occupational health nurse.

## 2. Materials and Methods

### 2.1. Design

This study used a qualitative content analysis method through a series of face-to-face and focus group interviews with workers, the employer, and the occupational health nurse to explore the contextual aspects of CVD risk factors perceived by workers in a small–medium-sized workplace. Various levels of factors suggested by the socioecological model that contributed to individual behaviors were theoretically linked to the data collection method (Table 1).

### 2.2. Participants

One small–medium-sized workplace comprising 117 workers, which manufactures audio equipment components in Seoul city, was selected for this study. The results of the workers’ regular health check-ups for the previous year were reviewed. Workers who were assessed to be at moderate or high risk according to the CVD risk-screening criteria proposed by the Korea Occupational Safety and Health Agency (KOSHA) [9] were considered potential study participants. A briefing session on the study was conducted with employer permission, and the researcher directly explained the study, including the purpose, methodology, and ethical protection of the participants, to 46 workers who participated in the session. Among the 36 workers who voluntarily engaged themselves to participate in this study, two workers with less than 1 year of experience were excluded as they were considered to have relatively insufficient exposure to the work environment and dynamic social experiences in the workplace. In total, 36 participants, consisting of 34 workers (19 individuals with a moderate risk, 13 individuals with a high risk), one health manager in charge of the workplace, and one visiting occupational health nurse, were selected as the final study participants.

### 2.3. Data Collection

Data for the contextual aspects of CVD risk factors in workers at an intrapersonal and organizational level were collected through individual face-to-face, in-depth interviews with workers, the employer, and the visiting occupational health nurse, who were all study participants. Data on the contextual aspects of CVD risk factors in workers at the interpersonal level were collected through the focus group interview method.

Individual data collection through in-depth interviews was conducted over approximately 5 months from December 2015 to April 2016 in a meeting room within the workplace and outside the working hours to prevent work interference. All interviews were recorded with prior participant consent, and lasted 20–45 min per person. Between one and three interviews were conducted for each participant, and additional interviews were conducted which is based on the need for data analysis. Interviews began with the key question, “what do you think are the contextual aspects that influence cardiovascular disease risks?”, followed by questions that were prepared and structured to be open for participants to disclose their thoughts and experiences with regard to the key question. In particular, the responses that identified influence from being a worker in a small–medium-sized workplace were investigated further to explore the essence of the participants’ response by asking participants to explain specific examples or situations. Interviews with the employer and occupational health nurse were conducted under the same conditions as the in-depth interviews with workers, and each interview began with the question “what do you think are the risk factors of the company which are associated with cardiovascular disease in workers from the employer’s point of view?” and “what do you think are the cardiovascular disease risk factors which influence the workers in a small–medium-sized workplace from the healthcare manager’s point of view?” for the employer and the nurse, respectively.

Furthermore, focus group interviews were conducted to explore the contextual aspects that influence CVD risks, interpersonally perceived by the workers. The degree of risk perception of CVD was divided into the high and low risk perception groups by using the question “How do you think your risk of cardiovascular disease is compared to peers of the same age and gender?” [10], and participants were divided into appropriate subgroups for focus group interviews. There were four groups, in total: two groups of nine participants with low risks of CVD, and two groups of eight participants with high risks of CVD. Pre-installed recording equipment at the interview site recorded the interviews conducted in accordance with pre-written interview guidelines. The open question “what influences the development of cardiovascular disease in the context of interactions between the employees?” allowed participants in the focus group to autonomously explore the essence of the issue in depth. Group interviews were conducted for approximately 45 min per group, and the researcher allowed the participants to talk freely while supporting all participants to express their opinions evenly.

The final questionnaires for data collection were completed after clarifying the purpose of the study based on advice from researchers of qualitative studies and literatures on study methods and the format and principles of research questions for both, in-depth face-to-face and focus group interviews.

### 2.4. Protection and Ethical Consideration of Participants

This study was approved by the Institutional Review Board at the researcher’s university (IRB No. KHSIRB-15-023). The researcher considered the entire study process to protect participant rights. During the recruitment process, the purpose of the study, process and frequency of the interview, the main questions, and the time required for the interviews were explained. In particular, the researcher explained that the utmost priority was for participants to engage voluntarily and that their interviews would be recorded, although the recorded content and field records would only be used for the sole purpose of this study, and that all records would be destroyed after the study. Interview materials containing information of individual workers would not be shared with the employer’s personnel, and participants could terminate their study engagement at any time without any disadvantages. After it was explained that all of the participants’ personal information would be treated anonymously and no personal information or private statements will be revealed in the results of this study, participants signed the consent form. A copy of the signed consent form and study proposal was provided to each participant.

### 2.5. Analysis

All collected transcripts were analyzed inductively based on the method proposed by Elo and Kyngas [11]. Data collection and analysis were conducted almost simultaneously in sequential steps, and the researcher sought to obtain more specific anecdotes or events in the context of risk factors for CVD, which was the unit of analysis for this study. For conceptual data abstraction, categories were inductively derived through a series of coding processes as follows: First, there was a process of familiarizing and focusing on the data by repeated readings of the entire transcript and parts of the transcript several times. Second, key words and phrases such as key statements and metaphors, which contribute to conceptualization, were highlighted during the read. Third, this was followed by open coding which was performed through line-by-line analysis. Fourth, a coding paper was produced by comparing and confirming the consistency of coding vocabulary, similarities, and differences in discussion with the research team. Fifth, specific codes were divided into subcategories. Sixth, the process of abstraction to a more comprehensive and higher category from the subcategories was done by identifying key statements, types, and attributes within and between the categories. Moreover, analytic memos were recorded after each interview throughout the research period and incorporated into the results.

### 2.6. Enhancing the Rigor of the Study

This study applied the strategy proposed by Shenton [12] to ensure rigor in qualitative research by following the criteria for assessing credibility, transferability, dependability, and confirmability presented by Guba [13] as the standards to ensure the rigor of qualitative research. Credibility refers to the internal validity of quantitative research, which indicates how accurately the results of the study describe and interpret the actual phenomena. Herein, the researcher aimed to increase the credibility of this study by collecting data over a prolonged period of several months, and by verifying the results of the data analysis with the main participants to examine whether the results reflect the authentic intentions and experiences of the participants. Transferability refers to external validity in quantitative research and indicates whether the results of the research can be applied to other situations. In this study, the meaning of a small workplace was clearly defined based on prior literature review, and the characteristics of the small workplace and the general characteristics of workers in the small workplace were described in detail. In addition, the results of the analysis were shared with an occupational health nurse who did not participate in this study to confirm whether the results could be applied to future research phenomena to explain the contextual aspects of CVD risks in workers at small workplaces. Dependability refers to the reliability of quantitative research, which indicates that similar results should be obtained if the study is conducted using the same method in the same population group. The researcher of this study ensured that future application of methods and procedures were possible by specifically explaining the processes of selection of participants, data collection, and data analysis. Confirmability refers to objectivity of the research, which outlines that the researcher’s individual bias was excluded from the research process and results. An audit trail was used to ensure the objectivity of this qualitative research. For this purpose, all possible information on the research data, including the time of collection, the content of the actions, and the results, were recorded using the researcher’s journal, photography, and recording devices. In addition, the researcher made every effort to ensure objectivity and exclude personal prejudice by sharing opinions on the data and qualitatively analyzing the data with researchers in regular meetings.

## 3. Results

### 3.1. General Characteristics of Study Participants

Table 2 depicts the general characteristics of 34 workers who were study participants, excluding the employer and the occupational health nurse. The majority of study participants were men (82.4%), and more than half of the participants were in their 40 s and 50 s (67.4%). In terms of education levels, 55.8% had education below high school. The majority of the employment was full-time, with an average of 5.3 years of work experience, and 52.9% of the employees had more than 5 years of experience. The percentage of smokers was 55.9%, which was higher than that of non-smokers, and 58.8% reported a family history of CVD such as stroke, angina, myocardial infarction that occurred before age 50 s. The level of triglyceride, blood pressure, BMI and fasting blood glucose were higher than the normal range.

### 3.2. Results of Content Analysis

Altogether 162 meaningful statements were investigated, and 27 sub-categories and 10 general categories were derived, the main categories were explored on the basis of the derived categories (Table 3). At the intrapersonal level, physical, cognitive, and psychological contextual risks were identified, whereas at the interpersonal level, social and environmental contextual risks were noted. At the organizational level, we investigated organizational vulnerability as a contextual factor that influences CVD risks in workers employed in small–medium-sized workplaces.

#### 3.2.1. Intrapersonal Physical, Cognitive, and Psychological Risks

Risk factors for cardiovascular disease at the intrapersonal level perceived by workers in small–medium-sized workplaces included physical burden due to physical labor, altered lifestyle habits due to irregular overtime work, lack of recognition and knowledge of CVD risks, and the priority for financial values over health as the head of the household with overburdening financial responsibilities.

##### Physical Burden

Study participants who were workers and had a main area of work in manufacturing considered physical fatigue caused by increased physical labor due to incessant physical work and lack of human resources as a risk factor for CVD.

*“If our job is to make and ship things right away, we work all the month, without Saturdays or Sundays, and we will work excessively. After working by using our bodies for a whole month, our bodies become very fatigued, so whether it is blood pressure or sugar, that’s beyond our control.”*

*(P1, male, 46 years old)*

##### Burnout Due to Overtime Work

Due to the nature of small–medium-sized workplaces, which frequently require overtime work according to production orders, participants were tired of irregular lifestyles. In particular, participants stated that they were compelled to be at risk of CVD due to overconsumption of calories from late-night meals, poor sleep quality, lack of sleeping time, and lack of exercise.

*“When I arrive home after working overtime, it’s about 12 o’clock in the morning. I have to wake up at 6:30 to go to work in the morning … then I really do not get enough sleep. So my blood pressure is bound to be high, isn’t it?”*

*(P33, female, 44 years old)*

##### Lack of Recognition and Knowledge of CVD Risks

This study was conducted among workers, who were at risk for CVD, but the participants had never heard of CVDs nor had any interest or knowledge of CVDs. Participants mentioned they were unaware of risks and management options for CVDs. Even when participants were aware of the risks, they considered it as an inevitable disease due to family history, or a disease, which occurs from the natural process of aging and, therefore, neglected management.

*“In fact, I am quite old, and most people in my age have one or two problems. I get high blood pressure and my sugar levels go up… I’m old, so no matter how much I care and live cautiously, my body still tends to get damaged somewhere I can’t help it.”*

*(P6, male, 51 years old)*

##### No Time to Take Care of Health Due to Family Responsibilities

Workers in their 40 s and 50 s comprised over half of the participants and said they must focus on earning more to make a living due to their current financial hardships, and their responsibilities as heads of the household. Thus, financial problems were prioritized over health concerns, including CVD.

*“I need to earn money. I don’t have time to manage my health. A lot of money needed is to raise children and I don’t have time to leisurely play sports and care for my health. We’re at the age to be working hard to support our children’s studies. If I could afford it financially, it would be good to care for my health as I am getting older, but I don’t have the financial leisure or the time for that.”*

*(P30, male, 48 years old)*

#### 3.2.2. Socio-Relational and Environmental Risks

Our investigation of the contextual aspects that influence CVD risks in interpersonal relationships perceived by the workers through focus group interviews indicated that the low-risk perception group prioritized financial issues over health issues and identified the health problems associated with CVD to be solely the individual’s fault or expressed fears of being stigmatized as an unhealthy person. In contrast, the high-risk perception group demonstrated a deeper understanding of CVD by specifically discussing experiences of job stresses from the relationship with their managers, and the vulnerable environment at work associated with CVDs. Both groups discussed the contextual aspects of CVD risks at the interpersonal level, as well as the perceived contextual aspects of CVD risks in the organizational environment. Results of specific analysis categorized by the two groups are as follows:

##### Low-Risk Perception Group

A. Stress of possible job losses

The low-risk perception group described that the stress from job insecurity caused by economic instability as a threat to their cardiovascular health.

*“A small company usually don’t have much funds, so at times like this, I’m worried that something might go wrong in the company. Our company is in the same boat, but every time people talk about it, I think I wonder if I will be the first to be fired, and then I can’t sleep… I think my blood pressure keeps rising because of that anxiety. Considering such things, this is not really the atmosphere to leisurely quibble about this (health management).”*

*(P22, male, 44 years old)*

B. Fear of being stigmatized as an unhealthy person

The low-risk perception group deliberately avoided or rejected healthcare services at the company from their fear of being stigmatized by colleagues and supervisors as an employee with health problems, and wanted to be considered by their colleagues and supervisors as individuals who do not require healthcare management. The anxiety that health problems could lead to job loss created mental stresses. Such workers thought that they did not need physical examinations, and expressed their usual disinterest in healthcare. In addition, some workers emphasized that CVDs were caused solely by the individual and responded they had no experience with CVD risk factors at the interpersonal level.

*“They don’t call for others but always call us gathered here separately, so it feels like I’m being called out because I really am sick somewhere… when the (occupational health) nurse calls me and I return after getting my blood pressure and sugar levels checked, I feel like people look at me strangely. Sometimes when people ask me if I am okay, all I have is a little high blood pressure and nothing contagious, but I feel offended because it feels like others are looking at me strangely. I feel like I may be stigmatized by the manager or the superiors as someone with a disease, so I feel uncomfortable. So there were often times when I did not go to the nurse on purpose, even though I was called.”*

*(P12, woman, 53 years old)*

##### High-Risk Perception Group

A. Smoking and drinking culture

Participants in the high-risk perception group perceived that stress caused by communication and interpersonal relationships with their managers was a risk factor for CVD, which was unlike the form of stress expressed by the low-risk perception group, which described job insecurity as a cause of workplace stress. In order to relieve such stress, workers stated that they smoked, had late-night meals, and drank together after work, to share their concerns and comfort each other. Above all, the workers in the high-risk perception group knew the significant impact of the drinking and smoking culture on the development of CVD. However, such culture was unavoidably generated as it was a form of stress relief despite awareness of harm to their health, and they concluded it was a group culture that they were compelled to continue.

*“I know this is a problem (night meal, alcohol drinking culture), but this is actually one of the ways for me to relieve my job stress. We talk together as we eat at night, talk behind our manager’s back, share if anything offended us, reconcile, or be comforted as we talk, and give each other courage. I know it’s a health problem to gather together after working overtime to eat and have a drink, but I don’t want to give it up.”*

*(P10, male, 42 years old)*

B. Work environment which does not promote healthy diet and rest

Workers in small businesses who often ate three meals at work rather than at home explained that the cafeteria in the workplace was operated without a specialized dietician. Consequently, the food was low in quality, salty, and spicy, and it was difficult for workers to manage their diet for CVDs. Furthermore, workers mentioned the vulnerability of the workplace environment in helping workers to relieve physical burden as workplaces did not have sufficient resting areas to relieve physical fatigue.

*“I have high blood pressure and sugar levels, so I cannot eat salty food and I have to eat lots of vegetables, but I can’t care for all those things when I’m working. Sometimes I eat all three meals at the company’s cafeteria, and it does not insufficiently provide nutrition. The food at the company’s cafeteria is salty, spicy, and strong because it is not made by experts who care about nutrition.”*

*(P26, female, 51)*

##### Organizational Vulnerability

The employer expressed occupational health services were unsatisfactory compared to the cost, and stated unwillingness to prioritize economic benefits of the company over workers’ health. The occupational health nurse mentioned the employer’s lack of interest in the importance of managing health for CVDs, and difficulties encountered in the process of obtaining management cooperation, such as securing a time and place to provide healthcare services.

A. Contextual risks perceived by the employer: Priority of company’s profits

The employer talked about the working environment, compelled to enforce sacrifices from workers due to the economic difficulties of the company; therefore, the employer was inclined to pursue company profits over worker health.

*“I also want to give my employees many holiday breaks, raise their wages, and care more for their health, but all these things are related to money, so how can this small company consider and provide all those things? It frustrates and aches my heart too… but what can I do? The company needs to survive first, to care for the employees’ health. No matter how healthy the workers are, if their workplace disappears, that is bigger trouble.”*

B. Contextual risks perceived by the visiting occupational health nurse: Lack of attention from the employer

The visiting occupational health nurse emphasized that employers in small workplaces are more interested in short-term and visible healthcare services, such as musculoskeletal disease management than CVD management, which requires long-term intervention. In other words, the biggest problem perceived by the nurse was the employer’s lack of interest and understanding of the management of CVDs. Moreover, the visiting occupational health nurse mentioned the experiences of limitations in providing services due to the difficulty in obtaining management cooperation for a time and place to provide health education for the management of CVDs.

*“When bones or muscles are damaged, then the workers who use their bodies to work must immediately stop working. Then it’s fatal. Honestly speaking, high blood pressure and high blood sugar levels does not mean that they must immediately stop working. Therefore, the concern of owners who operate small businesses are bound to be in the musculoskeletal diseases, rather than the management of cardiovascular diseases.”*

## 4. Discussion

This study was conducted to explore the contextual aspects that influence CVD risks perceived by workers, the employer, and the visiting occupational health nurse, who are the members of small–medium-sized workplaces based on the socioecological model.

The general categories of “physical burden” and “burnout due to overtime work” derived at the intrapersonal level were similar to “lack of time for healthcare” and “physical fatigue” resulted by “working overtime” explored in the previous studies [14,15] who applied qualitative research methods to explore the concerns and health needs of blue-collar workers. Small–medium-sized businesses, which are financially unstable and lack financial resources compared to large-scale businesses, make employees work overtime as the easiest method to reach the required workload; in order to replace their scarce manpower, the workload is increased as the company forces one worker to manage various roles, which results in physical and psychological burden [16]. These factors cause health problems such as fatigue, hypertension, sleep disturbance, and depression, which increase the risk of CVDs. In particular, South Korea has the second longest working hours among OECD countries after Japan, and small–medium-sized businesses with less than 300 employees constitute for 99.8% of all workplaces [2], with high risk of CVD from working overtime. Therefore, in order to prevent the development of CVD risks caused by working overtime for workers in small–medium-sized workplaces, agreements are necessary to provide financial support and rationally adjust working hours for small–medium-sized workplaces.

Previous studies have found that the socioeconomic status of blue-collar workers in small-sized businesses is relatively lower than in white-collar workers, and that the groups in the higher socioeconomic status enjoyed better diet and health benefits than vulnerable groups with lower socioeconomic status, suggesting that the higher socioeconomic status group had less exposure to CVD risk [17,18]. In this study, however, the workers in small–medium-sized workplaces overlooked the importance of health management for themselves because their priority was on financial values rather than health management for CVD, due to their low income and family responsibilities. In addition, the participants accepted CVDs as a natural consequence of aging and did not correctly recognize the importance of health management with the progression of age. This was similar to a previous study which identified that participants with coronary artery disease perceived “aging” as a risk factor for coronary artery diseases, but also perceived this as the process of “normalization” that occurs with age [19]. Furthermore, participants in this study recognized CVD as inevitable diseases caused by family history or heredity, which was similar to the results of a qualitative study of 38 women over 40 years of age that investigated their perception of contextual aspects, which act as threats for CVD risks [20]. The findings identified that participants accepted CVD as a disease of “fatalism”, which was bound to occur due to family history and heredity. Therefore, it is considered urgent to lead a change in the workers’ perception of CVDs to recognize the importance of early detection and active management of chronic diseases. This can be achieved when the workers in small–medium-sized workplaces autonomously take CVD risks seriously and accept an in-depth and systematic education on the prevention and management of CVD risks that increase with age.

At the intrapersonal level, the perceived contextual risks varied according to the degree of risks for CVDs. In the low-risk perception group, the contextual risks for CVD were perceived as a health problem that can be controlled by the individual, rather than a risk, which occurs in the context of interaction between employees. On the other hand, the high-risk perception group discussed work environmental factors, which cannot be controlled by individuals, and the two groups thereby showed differences in the perception of contextual aspects associated with CVD risks. Such findings in this study can be considered in relation to the optimistic bias [8,21,22], which occurs in individual during the perception of disease risks. In other words, individuals perceive themselves to be at lower risk of disease than others by adjusting their own risk factors for health and individuals thereby perform fewer preventative behaviors for their health [21,22,23,24,25]. Likewise, the low-risk perception group in this study perceived CVD risk factors to be caused by personal factors, which can be controlled by the individual rather than as factors, which arise from interactions with others in interpersonal relationships, which demonstrated the sociopsychological trends of optimistic bias towards cardiocerebrovascular disease risks. In particular, optimistic bias appears to be motivated by self-defense against negative events and to reduce anxiety [23,25], which makes individuals control risk factors on their own to decide that they can be healthier [23,24,25]. Likewise, the low-risk perception group in this study perceived anxiety related to potential unemployment from stigmatization as an unhealthy person as a CVD risk factor and chose to avoid health checks as a method of controlling the issue to demonstrate to colleagues and managers that they were healthy. The results of this study showed that the optimistic bias derived from such actions tended to lower the group’s perception of CVD risks, which corresponded to the results of the previous studies. The optimistic bias phenomena have been found even recently in health-related fields such as smoking [26], cancer [27], and CVD [28] and has been a barrier in raising awareness of disease risks through health promotion programs and health campaigns, and in encouraging action to prevent diseases [27,28,29]. Therefore, when developing and applying health promotion programs to prevent CVD in workers from small workplaces, the measures to confirm and control obstacles such as optimistic bias at the intrapersonal level during the planning phase must be identified, to maximize the effectiveness of the programs.

On the other hand, the perception of risk factors towards the smoking and alcohol drinking culture in the high-risk perception group led to the recognition that such factors cannot be controlled by individual behavior, which made a difficulty in the group to practice healthy behaviors. This was similar to the results of a qualitative study on the perception of CVD risks in middle-aged males with acute myocardial infarction [28]. The study identified that the participants were compelled to smoke to relieve the stress from their jobs, although smoking was perceived as a cause of myocardial infarction. Encouraging workers who spend most of their day at work to be aware of the risks arising from their relationships with colleagues at the interpersonal level and encouraging interactions that promote each other’s health behaviors is vital to improve health in the workplace. However, according to a systematic study of the intervention to prevent CVD in workers conducted from an ecological perspective [5], the number of studies, which analyzed risk factors at the interpersonal level, including subsequent intervention studies, were only three, which was the least number of studies conducted as compared to interventional studies at other levels. This is due to the lack of cooperation by employers when conducting interventional studies at a workplace, which makes it challenging to secure a time and place for group lectures or group discussions on the workers’ health management, and due to the lack of interest from workers and employers in healthcare intervention programs [29], which is at the interpersonal level, and most essentially requires interaction and collaboration, identified to be difficult. Therefore, it is necessary to develop and apply interventional studies to promote awareness of the risk factors occurring from interactions with colleagues which otherwise act as threats for CVDs, and to promote healthy behaviors in workers of small–medium-sized workplaces. In addition, interventional studies, including interventions at the organizational level, should be conducted to reduce workers’ job stress and create a healthy workplace culture that can replace the smoking and drinking culture.

Lastly, at the organizational level, the employer stated that the company’s profit pursuit is given priority over workers’ health due to the characteristics of a small–medium-sized workplace, which concludes with the theme “the company’s benefits come first”. In order to maintain the business, the healthcare manager stated there were difficulties in proceeding with the project due to lack of interest from the employer in the health management. This is consistent with the results from the nursing activity experience study conducted as a health technology support project for small businesses in 2002 [29], which outlined that the priority was placed in the company’s interests. Such results indicated a continuing need for change in the employers’ interests and perceptions of cardiovascular diseases. The passive attitude of employers towards health management is due to the lack of short-term and effective impact on corporate productivity, and the perception that health management must be long-term and is time-consuming [30]. Furthermore, such passive attitude can cause deterioration in the workers’ health from CVDs, which trigger economic loss at the organizational and social levels in the long term. Therefore, occupational health nurses must share information and raise awareness of health management by frequently meeting with employers of the small–medium-sized workplaces. This requires regulation supports to ensure that workers meet regularly with occupational health nurses for it. In addition, occupational health nurses should establish and provide analysis, which depicts the cost effectiveness of the company with healthy workers and the data on economic loss of the company due to workers’ illnesses. Furthermore, the occupational health nurses must encourage the employers of small businesses to participate more actively in health promotional projects for employees at a level that corresponds with the economic value expected by the employers in small businesses.

The ecological model applied in this study is useful for healthcare studies as it can analyzed, understood, and reach a stage of mediating risk factors through a multidimensional approach, from the individual level to the organizational level, to affect the individual’s behavior for health. However, due to the practical constraints, it is difficult to expand the study to the community level or apply the study to the policy level, which is a limitation that remains as a challenge for health professionals. In addition, the limitations of this study were evident, as the risk factors at the community and policy levels, from the five dimensions presented in the ecological model, were not explored, despite endeavoring to explore the contextual aspects that affect cardiovascular diseases risks in workers from small–medium-sized workplaces through a multidimensional approach based on the ecological perspective. Moreover, the proportion of employers and healthcare managers was relatively small compared to the number of workers in the study, which was an obstacle in sufficiently drawing the practical contextual aspects of cardiovascular disease risks in workers perceived by employers and healthcare managers, at the organizational level, in small–medium-sized workplaces. Despite the limitation that not all levels present in the model were applied, this study is nevertheless significant as it explored the contextual aspects that influence the CVD risks perceived by members in the workplace, and the differences in the degree of workers’ perception of risks by using qualitative research methods which have not been discussed in the prior literature regarding the factors affecting CVDs in workers from small–medium-sized workplaces, and for providing discussions with a practical approaches to provide evidence for occupational healthcare professionals to identify effective methods for intervention.

The following suggestions are proposed based on this study: First, follow-up studies with larger number of participants is suggested to sufficiently draw the contextual risks of CVD in workers from small–medium-sized workplaces, which are perceived by the employers and occupational health nurses at the organizational level. Second, it is suggested to conduct additional research, which identifies contextual risk factors affecting CVD in small workplace workers at the extended community and policy levels. Third, previous study suggests that participant-centered intervention methodologies such as participatory action research (PAR) and Participatory Action-Oriented Training (PAOT) in the workplace as well as qualitative research method could be the effective health promotion programs to improve health-related self-regulation and health-promoting behavior for workers [18]. Therefore, additional PAR or PAOT intervention studies should be conducted to elucidate the effect of participant-centered methodologies on reducing CVD risk for workers in small–medium-sized workplaces. Fourth, this study confirmed that recognized risk context for CVD differs according to the degree of risk perception. In other words, it means that the degree of disease perception can act as a covariate that can affect health promotion behaviors in an intervention study. Therefore, when applying an intervention study for health promotion, it is necessary to clarify the research design and statistical analysis methods that can control covariates.

## 5. Conclusions

The contextual aspects, which affect workers in small–medium-sized workplaces with CVD risks were investigated based on an ecological perspective to prevent CVDs, and to provide evidence for effective CVD management in workers from small–medium-sized workplaces. Workers in small–medium-sized workplaces were identified to have perceptions and experiences of contextual aspects, which affect cardiovascular disease risks at the intrapersonal, interpersonal, and organizational levels, and the experiences of contextual risks for cardiovascular disease varied according to the degree of risk perception of the disease. Therefore, when developing health programs for CVDs in workers from small–medium-sized workplaces in the future, a comprehensive consideration of the contextual risk factors perceived and experienced by the workers in the workplace at the intrapersonal, interpersonal, and organizational levels is required, and the focus should be to improve such factors. In particular, interventional methods should be developed and applied by considering the differences in the degree of the individual’s perception of disease risks. Furthermore, the employers’ perception of healthcare programs must be improved and proactive attention from the occupational health industry must be rendered to increase the effectiveness of the programs.

## Figures and Tables

**Table 1:** Data-collection procedure based on the socioecological model.

Ecological Model	Methods	
Intrapersonal Level	Individual interviews (*n* = 34)	
Interpersonal Level	Focus Group Interviews ^†^ Group A-1 (*n* = 9), Group A-2 (*n* = 9) ^‡^ Group B-1 (*n* = 8), Group B-2 (*n* = 8)	
Organizational Level	Individual (Key persons: health manager and occupational health nurse)) interviews (*n* = 2)	

**^†^** Group A: Low risk perception, **^‡^** Group B: High risk perception.

**Table 2:** General characteristics of the study population (*n* = 34).

Variables	Total *n* (%)/Mean (SD)
Sex	
Male	28 (82.4)
Female	6 (17.6)
Age (year)	42.8 (7.1)
20–39	11 (32.4)
40–59	23 (67.4)
Educational level	
≤High school	19 (55.8)
≥College	15 (44.2)
Marital status	
Married	23 (67.6)
Unmarried	11 (32.4)
Employment	
Regular	29 (80.6)
Contract	5 (13.9)
Job tenure (year)	
2–5	16 (47.1)
>5	18 (52.9)
Smoking	
Non-smoker	10 (29.4)
Ex-smoker	5 (14.7)
Smoker	19 (55.9)
Total cholesterol (mg/dL)	204.7 (44.1)
Low density lipoprotein cholesterol (mg/dL)	126.8 (47.4)
High density lipoprotein cholesterol (mg/dL)	55.7 (30.7)
Triglyceride (mg/dL)	246.7 (213.2)
Fasting blood glucose (mg/dL)	108.8 (35.4)
Body Mass Index (kg/m^2^)	26.2 (5.1)
Normal weight	8 (23.5)
Overweight	8 (23.5)
Obesity	18 (52.9)
Systolic blood pressure (mmHg)	135.9 (13.39)
Diastolic blood pressure (mmHg)	88.9 (8.4)
Family history related to cardiovascular disease	
No	20 (58.8)
Yes	14 (41.2)
Stroke	4 (28.6)
Angina	5 (12.8)
Myocardial Infarction	5 (44.6)

**Table 3:** Overview of categories and themes for CVD risk contexts.

Main Themes	Generic Category		Sub-Category
Physical, cognitive, and psychological risks	Physical burden		Physical burden because of physical labor
			Increased physical labor because of the lack of human resources
			Fatigue caused by frequent overtime work
	Burnout due to the overtime work		Lack of leisure activities
			An irregular life
			Late-night meal (snack)
			Lack of exercise
			Lack of sleep
	Lack of recognition and knowledge of CVD risks		Inevitable consequence caused by family history
			Inevitable consequence caused by aging
			Believe that have no health problems.
			No interest in CVD
			Lack of CVD knowledge
	No time to take care of health because of family responsibility		Burdensome duties as a patriarch
			Have to work to make money for families
Socio-relational and environmental risk	Low risk perception	Stress of possible job losses	Increased anxiety due to company instability
		Fear of being stigmatized as an unhealthy person	Avoided health check-up because of possibly being stigmatized as an unhealthy person
			Health problems attributed to individual factors
	High risk perception	Smoking and drinking culture	Smoking and drinking culture as job stress-relief groups
			Communication stress in relationship with a supervisor
		Work environment which does not promote healthy diet and rest	Unhealthy menu in cafeteria
			Lack of resting places
Organizational vulnerability	Employer	Priority of company’s profits	Unsatisfactory for the occupational health services
			Preferred economic value than employees’ health
	Occupational health nurse	Lack of attention from the employer	Lack of interest of employer for the employees’ CVD health
			Lack of coordinated time for the health services
			Lack of suitable place for health services
